# Global Genetic Determinants of Mitochondrial DNA Copy Number

**DOI:** 10.1371/journal.pone.0105242

**Published:** 2014-08-29

**Authors:** Hengshan Zhang, Keshav K. Singh

**Affiliations:** 1 Hengshan Zhang, Department of Central Laboratory, First Affiliated Hospital of Fujian Medical University, Fuzhou, China; 2 Departments of Genetics, Pathology, Environmental Health, Center for Free Radical Biology, Center for Aging and UAB Comprehensive Cancer Center, University of Alabama at Birmingham, Birmingham, Alabama, and Birmingham Veterans Affairs Medical Center, Birmingham, Alabama, United States of America; UMASS-Amherst/Tufts University School of Medicine, United States of America

## Abstract

Many human diseases including development of cancer is associated with depletion of mitochondrial DNA (mtDNA) content. These diseases are collectively described as mitochondrial DNA depletion syndrome (MDS). High similarity between yeast and human mitochondria allows genomic study of the budding yeast to be used to identify human disease genes. In this study, we systematically screened the pre-existing respiratory-deficient *Saccharomyces cerevisiae* yeast strains using fluorescent microscopy and identified 102 nuclear genes whose deletions result in a complete mtDNA loss, of which 52 are not reported previously. Strikingly, these genes mainly encode protein products involved in mitochondrial protein biosynthesis process (54.9%). The rest of these genes either encode protein products associated with nucleic acid metabolism (14.7%), oxidative phosphorylation (3.9%), or other protein products (13.7%) responsible for bud-site selection, mitochondrial intermembrane space protein import, assembly of cytochrome-c oxidase, vacuolar protein sorting, protein-nucleus import, calcium-mediated signaling, heme biosynthesis and iron homeostasis. Thirteen (12.7%) of the genes encode proteins of unknown function. We identified human orthologs of these genes, conducted the interaction between the gene products and linked them to human mitochondrial disorders and other pathologies. In addition, we screened for genes whose defects affect the nuclear genome integrity. Our data provide a systematic view of the nuclear genes involved in maintenance of mitochondrial DNA. Together, our studies i) provide a global view of the genes regulating mtDNA content; ii) provide compelling new evidence toward understanding novel mechanism involved in mitochondrial genome maintenance and iii) provide useful clues in understanding human diseases in which mitochondrial defect and in particular depletion of mitochondrial genome plays a critical role.

## Introduction

Mitochondria are the sites of vital cellular functions such as the synthesis of the cellular energy by oxidative phosphorylation (OXPHOS), the tricarboxylic acid (TCA) cycle, amino acid, and heme biosynthesis [1]. Deleterious mitochondrial DNA (mtDNA) mutations have been reported to cause a broad spectrum of cellular and organismal responses including apoptosis, carcinogenesis, aging and neurological degeneration, and are an important cause of inherited disease. For example, mtDNA missense mutations have been associated with ophthalmological and neurological diseases known as Leber's Hereditary Optic Neuropathy (LHON), and Neurogenic muscle weakness, Ataxia and Retinitis Pigmentosa (NARP). Transfer RNA mutations are now known for Myoclonic Epilepsy and Ragged-Red Fiber Disease (MERRF); Mitochondrial Encephalomyopathy, Lactic Acidosis, and Strokelike Symptoms (MELAS); and Maternally Inherited Myopathy and Cardiomyopathy (MMC). Another frequently described pathology associated with reduced mtDNA content is mtDNA depletion syndromes (MDS), a group of severe diseases of childhood. The affected persons usually suffer from quantitative decrease in copy number of mtDNA with progressive pathophysiological disorders and subsequent death in their first year of life [2], [3]. To date, over 100 heritable disorders have been attributed to defects in mitochondrial function in humans, and new mitochondrial disease genes are still being identified [4].

Human mtDNA contains one single control region called the D-loop that controls mtDNA replication and transcription of mtDNA-encoded OXPHOS genes, Mutation in the D-loop region is a universal feature and has been reported in all tumors examined to date [5]–[7]. Mutations in the D-loop region result in altered binding affinities of the nuclear proteins involved in mtDNA replication and transcription leading to the depletion of mtDNA content [8]. Consistent with this notion, our laboratory recently reported a near absence of mtDNA-encoded cytchrome c-oxidase subunit II expression in more than 40% of breast and ovarian tumors [9]. Other laboratories measured mtDNA directly in paired normal and tumors and reported a decrease in mtDNA content in breast [10], [11], renal [12], hepatocellular [13], [14], gastric [15] and prostate tumors [16]. Reduced mtDNA is shown to decrease mtOXPHOS activity in renal tumors [17]. A study also demonstrated that decrease in mtDNA content correlates with tumor progression and prognosis in breast cancer patients [18]. Indeed our studies demonstrate that reduced mtDNA content leads to tumorigenic phenotype *in vitro* [19]. These studies suggest that mtDNA homeostasis plays an important role in tumorigenesis and may contribute to Warburg effect.

The maintenance of functional mitochondria in yeast and higher eukaryotic cells is governed by genes present in mitochondrial DNA and genes located in chromosomal DNA [20]. Human mtDNA encodes 13 polypeptide components of the respiratory-chain enzyme complexes (Complex I, III, IV and ATP synthase) and houses genes specifying 2 rRNA and 22 tRNA that are components of mitochondrial proteins synthesis system [21]. All of the remaining over 98% mitochondrial components are encoded by nuclear genes and targeted to the organelle by specific mitochondrial import systems. These include majority of OXPHOS proteins, the metabolic enzymes, the DNA and RNA polymerases, the ribosomal proteins, and the mtDNA regulatory factors [22]. Although mitochondria possess their own translational machinery, they largely depend on these proteins to fulfill their functions. In either mitochondria or cytoplasm, protein synthesis takes place on ribosomal subunits, where the genetic information transcribed into mRNA is translated into protein. Mitochondrial ribosomal RNAs which together with mitochondrial ribosomal proteins (MRPs) are components of ribosomal subunits. The genes for mitochondrial ribosomal proteins are mostly encoded in the nuclear genome [23]–[25]. The process of protein synthesis on the ribosome consists of three phases including initiation, elongation and termination.

So far, our knowledge concerning the genes that are involved in the maintenance of mitochondrial genome is incomplete [26], [27], and little is known about the nuclear genes and pathways whose inactivation lead to a loss or reduction of mtDNA [28]–[30]. The budding yeast *Saccharomyces cerevisiae* can grow and divide in the absence of respiration or mtDNA, and its mitochondrion is a highly functionally similar with human mitochondria, providing a particularly suitable model for identifying nuclear gene involvement in the maintenance of the mitochondrial genome. We screened respiratory-deficient *S. cerevisiae* strains, which belong to class III strains identified by Steinmetz and coworkers recently using the whole-genome pool of yeast deletion mutants [31]. Importantly, our analyses led to identification of a total number of 102 nuclear genes whose absence results in a complete loss of mtDNA, 52 of which are not reported previously. We also extended our effort to find human orthologs of the identified yeast genes and analyzed the possible roles of the orthologs in human diseases. Additionally, we performed *CAN1* forward-mutation assay on the yeast mutants in an effort to screen for genes whose defect affect the integrity of nuclear genome in yeast.

## Materials and Methods

### Yeast strains, media and reagents

The yeast strains used in this study were wild-type BY4741 (*MATa his3Δ1 leu2Δ0 met15Δ0 ura3Δ0*) and its 466 haploid deletion mutants with deficiencies in growth on non-fermentable substrates [31]. The strains were grown in standard media including yeast extract/peptone/dextrose (YPD) medium or synthetic complete medium (SC) lacking the appropriate amino acid.

### Fluorescent microscopy

Patches of the wild-type and each individual deletion strain were made on YPD plates and incubated at 30°C for two days. Cells were picked up and spread across a clean glass slide after blended with 10 µl of H_2_O. Cells were then air-dried and fixed with methanol and acetic acid (3∶1) for 15 minutes at room temperature followed by washing twice in PBS. Cells were again air-dried and then stained with the VECTASHIFLD mounting media with DAPI (1.5 µg/ml, Vector Laboratories, CA.) for 15 minutes at room temperature. Cells were visualized using an optimized filter under Nikon microscope with FISHView system (Applied Spectral Imaging, Inc. Carlsbad, CA) and images were captured with a CCD camera.

### Interaction analysis of identified genes

Yeast genes of interest were screened for interaction with the program Pathway Assist (version 3.0, Stratagene Corp. La Jolla, CA), and interactions among these genes were analyzed. Pathway Assist is a software tool for biological pathway analysis. It allows for the identification and visualization of pathways, gene regulation networks and protein interaction maps. The software program utilizes a natural language processor to extract information from databases such as Pub Med to provide direct associations.

### Nuclear mutation spectra

The wild-type strain BY4741 and each individual deletion strain were grown in 96-well YPD plates at 30°C for 2 days. 5 µl aliquot of each of these cultures was then spoted on SC arginine-dropout plates containing 60 mg/liter canavanine and incubated for 4 days to detect the presence of canavanine-resistant mutants (Can^r^). Each spot was scored as follows: 0 for spots with 0–5 Can^r^ colonies, similar to the wild-type strain; + for spots with 6–10 colonies; ++ for spots with 11–15 colonies; +++ for spots with >16 colonies or confluent. All mutants with a score of + or greater were further tested by performing the *CAN1* forward mutation test by growing cells in 5 ml of YPD to saturation followed by plating an aliquot onto YPD and the SC arginine drop-out canavanine-containing plates after washing and diluting in sterilized water. The numbers of Can^r^ colonies with respect to viable cells were determined after 4–5 days at 30°C. These experiments were performed by growing five independent cellular isolates for each strain. CA*N1* gene of yeast encodes a transmembrane amino acid transporter that renders the cell sensitive to a lethal arginine anolog, canavanine. Any inactivating mutation in this gene results in a Can*^r^* phenotype. Thus, the frequency of Can*^r^* colonies measures spontaneous nuclear mutational events [32], [33].

### Statistics Analysis

Statistical analysis for the mutations frequency was performed using Student's *t*-test assuming unequal variance. In all cases, statistical significance was determined at the 0.05 level.

## Results

### Genetics screen for mtDNA depletion

**Figure 1:**
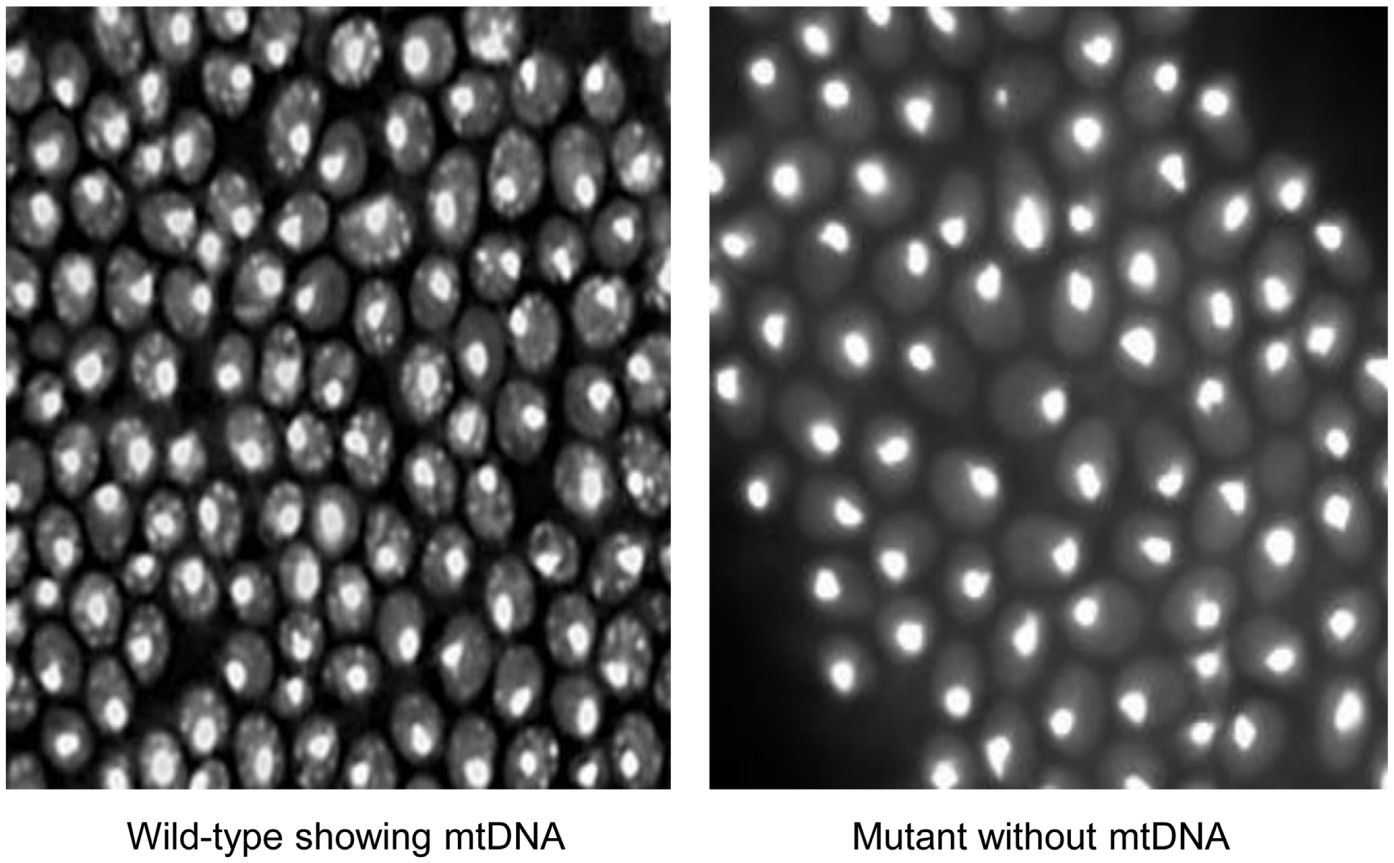
Representative image showing depletion of mtDNA. Cells were visualized after stained with DAPI (see Materials and Methods). Image on left and right panels show mitochondrial DNA profile in wild-type and mutant strain, respectively.

We performed a systematic screen of yeast mutants to identify genes whose deletions led to a complete loss of mtDNA. Among the respiratory defective strains screened, 102 were found to have no mtDNA, of which 52 were not reported previously. The screen was performed twice in a blind manner with regard to the identity of the deleted ORFs, and consistent results were obtained. The representative image profile of mtDNA depletion is shown in Figure 1.

### Defects in mitochondrial protein synthesis induce depletion of mtDNA

We found that 56 of the 102 yeast strains (54.9%) that lost their mtDNA were associated with deletions of genes which function in mitochondrial protein synthesis. It is shown in Table 1 that these genes encode 38 mitochondrial ribosomal proteins (37.3%), 9 aminoacyl-tRNA synthetases, amino acid activating enzymes (8.8%) and 7 protein products involved in mitochondrial ribosome recycling or assembly, mitochondrial translation elongation, peptide chain release, all of which are essential components for protein translation process. In addition, 1 of these genes encodes cytosolic 60S large subunit ribosomal protein.

**Table 1:** Genes involved in mitochondrial protein biosynthesis.

Gene deleted	Human ortholog	Wild-type function
*RML2*	*LOC51069*	Mitochondrial ribosomal protein L2 of the large subunit protein biosynthesis
*RSM18*		Component of the mitochondrial ribosomal small subunit, protein biosynthesis
*FIL1*		Mitochondrial ribosome recycling factor, protein biosynthesis
*MSR1*		Nuclear-encoded mitochondrial protein, arginyl-tRNA synthetase, protein biosynthesis
*MRPL8*	*LOC63875*	Mitochondrial ribosomal protein of the large subunit, protein biosynthesis, mitochondrial genome maintenance
*MEF2*		Mitochondrial elongation factor G-like protein, protein synthesis elongation
*MRPL24*		Mitochondrial ribosomal protein of the large subunit, Protein biosynthesis
*MRPL23*	*MRPL13*	Mitochondrial ribosomal protein of the large subunit Protein biosynthesis
*MSD1*	*FLJ10514*	Mitochondrial aspartyl-tRNA synthetase, protein biosynthesis
*MRP51*		Mitochondrial ribosomal protein of the small subunit, Protein biosynthesis
*MRPL40*		Mitochondrial ribosomal protein of the large subunit, Protein biosynthesis
*MRPL16*		Mitochondrail ribosomal protein of the large subunit, Protein biosynthesis
*MRP21*		Mitochondrial ribosomal protein, protein biosynthesis
*MRPS5*	*MRPS5*	Mitochondrial ribosomal protein S5, Protein biosynthesis
*MRPL37*		Mitochondrial ribosomal protein of the large subunit, Protein biosynthesis, mitochondrial
*MRPL11*		Mitochondrial ribosomal protein of the large subunit, protein biosynthesis
*MRPL32*		Mitochondrial ribosomal protein of the large subunit, Protein biosynthesis
*YDR115W*	*MRPL34*	Putative mitochondrial ribosomal protein of the large subunit, protein biosynthesis
*MRPL7*		Mitochondrial ribosomal protein of the large subunit, Protein biosynthesis
*MRPL35*	*PBP*	Mitochondrial ribosomal protein of the large subunit Protein biosynthesis
*MRPS28*		Mitochondrial ribosomal protein of the small subunit, Protein biosynthesis
*MRP20*	*MRPL23*	Mitochondrial ribosomal protein of the large subunit, Protein biosynthesis
*RPL1B*	*RPL10A*	Cytosolic 60S large subunit ribosomal protein, protein biosynthesis
*MRPL9*	*MRPL3*	Mitochondrial ribosomal protein of the large subunit, Protein biosynthesis
*MRPL25*		Mitochondrial ribosomal protein of the large subunit, Protein biosynthesis
*MRPL27*		Mitochondrial ribosomal protein of the large subunit, Protein biosynthesis
*YCR024C*	*FLJ23441*	Mitochondrial asn-tRNA synthetase, Protein biosynthesis
*MRPL17*	*C15orf4*	Mitochondrial ribosomal protein of the large subunit, Protein biosynthesis
*MRP7*		Mitochondrial ribosomal protein of the large subunit, Protein biosynthesis
*MSE1*		Nuclear-encoded mitochondrial protein, glutamyl-tRNA synthetase, protein biosynthesis
*MRP2*	*MRPS14*	Mitochondrial ribosomal protein of the small subunit, Protein biosynthesis
*MSF1*	*LOC91893*	Mitochondrial phenylahanine-tRNA ligase alpha chain, Protein biosynthesis
*MRPL20*		Mitochondrial ribosomal protein, protein biosynthesis
*MRPL38*		Mitochondrial ribosomal protein of the large subunit, Protein biosynthesis
*MRPL31*		Mitochondrial ribosomal protein, Protein biosynthesis
*MEF1*	*EFG*	Mitochondrial translation elongation factor G, Protein synthesis elongation
*MRPL6*		Mitochondrial ribosomal protein of the large subunit, Protein biosynthesis
*DIA4*	*FLJ20450*	Probable mitochondrial seryl-tRNA synthetases, Pseudohyphal growth
*TUF1*	*TUFM*	Mitochondrial translation elongation factor Tu
*MSY1*	*LOC51067*	Mitochondrial tyrosyl-tRNA synthetase
*MSW1*		Mitochondrial tryptophanyl-tRNA synthetase
*MRF1*		Mitochondrial peptide chain release factor, protein synthesis
*ISM1*	*FLJ10326*	Mitochondrial isoleucyl-tRNA synthetase
*RSM23*		Mitochondrial ribosomal protein of the small subunit
*RSM22*		Mitochondrial ribosomal protein of the small subunit, Protein biosynthesis
*RSM27*		Mitochondrial ribosomal protein of the small subunit, Protein biosynthesis
*RSM7*		Mitochondrial ribosomal protein of the small subunit, Protein biosynthesis
*MSK1*	*KARS*	Mitochondrial lysine-tRNA synthetase
*RSM19*		Mitochondrial ribosomal protein of the small subunit, Protein biosynthesis
*MTG1*		Peripheral GTPase of the mitochondrial inner membrane, protein biosynthesis and ribosome assembly
*MRPL51*		Mitochondrial ribosomal protein of the large subunit, Protein biosynthesis
*YNR036C*	*MRPS12*	Putative mitochondrial ribosomal protein of the small subunit, protein biosynthesis
*MRPS8*		Mitochondrial ribosomal protein of the small subunit, Protein biosynthesis
*MTG2*	*GTPBP5*	Mitochondrial GTP binding protein required for mitochondrial translation
*MRPS16*	*MRPS16*	Mitochondrial ribosomal protein of the small subunit, Protein biosynthesis
*YNL081C*		Putative mitochondrial ribosomal protein of the small subunit, Protein biosynthesis

### Defects in nucleic acid metabolism induce depletion of mtDNA

Among 102 no-mtDNA yeast strains, 15 (14.7%) were found to have deletions of genes involved in nucleic acid metabolism such as DNA or RNA polymerase transcription, DNA binding, replication or repair, one-carbon compound metabolism (Table 2).

**Table 2:** Genes involved in nucleic acid metabolism.

Gene deleted	*Human ortholog*	Wild-type function
*RPO41*		DNA-directed RNA polymerase, transcription from mitochondrial promoter
*MGM101*		DNA binding, mitochondrial genome maintenance
*ABF2*	*HMG4*	DNA binding, mitochondrial genome maintenance
*RNR4*	*RRM2*	Ribonucleotide reductase small subunit, DNA replication
*SSQ1*	HSPA9B	Mitochondrial heat shock protein 70, DNA dependent DNA replication
*MSH1*	*MSH3*	Mitochondrial DNA mismatch repair
*MIP1*		Mitochondrial DNA polymerase catalytic subunit, DNA- dependent DNA replication
*FZO1*		Mitochondrial integral membrane protein involved in mitochondrial fusion and maintenance of mitochondrial genome
*MET7*	FPGS	Tetrahydrofolylpolyglutamate synthase, required for methionine synthesis and for maintenance of mitochondrial DNA, involved in one-carbon compound metabolism
*PET56*	FLJ22578	Ribose methyltransferase for mitochondrial 21 S rRNA
*NAM2*	*KIAA0028*	Mitochondrial leucyl-tRNA synthetase, mRNA binding
*SUV3*	*SUPV3L1*	Mitochondrial ATP-dependent RNA helicase, RNA catabolism
*HMI1*		Mitochondrial inner membrane localized DNA helicase, maintenance of the mitochondrial genome
*AEP3*		Peripheral mitochondrial inner membrane protein, mRNA metabolism
*MRH4*	*U5-100K*	Mitochondrial RNA helicase, ribosome biogenesis

### mtDNA depletion resulting due to defects in oxidative phosphorylation

We also found that among the 102 identified mutants, 4 (3.9%) have disruptions of nuclear genes responsible for mitochondrial oxidative phosphorylation. These genes normally encode a component in F1F0-ATPase complex or a protein involved in the synthesis/assembly of iron-sulfur centers (Table 3).

**Table 3:** Genes involved in Oxidative Phosphorylation.

Gene deleted	Human ortholog	Wild-type function
*ATP5*	*ATP5O*	F1F0-ATPase complex, OSCP subunit, ATP synthesis coupled proton transport
*ATP4*	ATP5F1	F1F0-ATPase complex, F0 subunit B, ATP synthesis coupled proton transport
*ATP14*		F1F0-ATPase complex, subunit h, ATP synthesis coupled proton transport
*GRX5*	TXNL2	Mitochondrial matrix protein involved in the synthesis/assembly of iron-sulfur centers

### mtDNA depletion due to other cellular defects

In addition, the complete depletion of mtDNA in 14 (13.7%) strains stems from a variety of other defects including bud-site selection, mitochondrial intermembrane space protein import, assembly of cytochrome-c oxidase, vacuolar protein sorting, protein-nucleus import, calcium-mediated signaling, heme biosynthesis or iron homeostasis (Table 4).

**Table 4:** Genes involved in other biological processes.

Gene deleted	*Human ortholog*	Wild-type function
*BUD16*	*PDXK*	Involved in bud-site selection
*AFG3*		Component of the mitochondrial inner membrane m-AAA protease, mitochondrial intermembrane space protein import, assembly of mitochondrial enzyme complexes
*PET100*		Mitochondrial inner membrane chaperone that specifically facilitates the assembly of cytochrome-c oxidase
*VPS45*	*VPS45B*	Vacuolar protein sorting-associated protein, Golgi to vacuole transport
*KAP123*	*FLJ23338*	Protein-nucleus import
*MDJ1*	*TID1*	Protein involved in folding of mitochondrially synthesized proteins in the mitochondrial matrix, member of the DnaJ family of molecular chaperones
*LCB5*	SPHK1	Sphingolipid long chain base kinase, sphingolipid metabolism, calcium-mediated signaling
*YTA12*	*AFG3L2*	Mitochondrial intermembrane space protein import, Protein complex assembly
*EUG1*	GRP58	Protein disulfide isomerase, protein folding
*HEM14*	*PPOX*	Mitochondrial protoporphyrinogen oxidase, heme biosynthesis
*Oct1*	*MIPEP*	Mitochondrial intermediate peptidase, cleaves N-terminal residues of a subset of proteins upon import, iron homeostasis
*PPT2*		Phosphopantetheine: protein transferase, activates mitochondrial acyl carrier protein, lipid transport
*ISA2*		Protein required for maturation of mitochondrial and cytosolic Fe/S proteins, localizes to the mitochondrial intermembrane space, iron transport
*MTM1*	*LOC51629*	Putative mitochondrial carrier protein, manganese ion transport

### mtDNA depletion due to deletions of genes involved in unknown function

The remaining 13 yeast mutants (12.7%) identified lost their mtDNA completely due to the deletions of nuclear genes whose protein products are not yet established functionally (Table 5).

**Table 5:** Genes involved in unknown biological processes.

Gene deleted	Human ortholog	Wild-type function
*YGR102C*		Mitochondrial, biological process unknown
*YGR150C*		Mitochondrial, biological process unknown
*YPR116W*		Mitochondrial, biological process unknown
*YOR305W*		Mitochondrial, biological process unknown
*YLR091W*		Mitochondrial hypothetical protein, biological process unknown
*QRI5*		Mitochondrial protein of unknown function
*YOR199W*		Biological process unknown
*YOR205C*		Hypothetical protein, Biological process unknown
*QRI7*	LOC64172	Mitochondrial, biological process unknown
*YDR065W*		Mitochondrial hypothetical protein, biological process unknown
*YDR114C*		Biological process unknown
*CAF17*	LOC200205	Mitochondrial protein, biological function unknown
*SOV1*		Hypothetical protein, protein function unknown

### Genes involved in synthetic lethal without mtDNA

**Figure 2:**
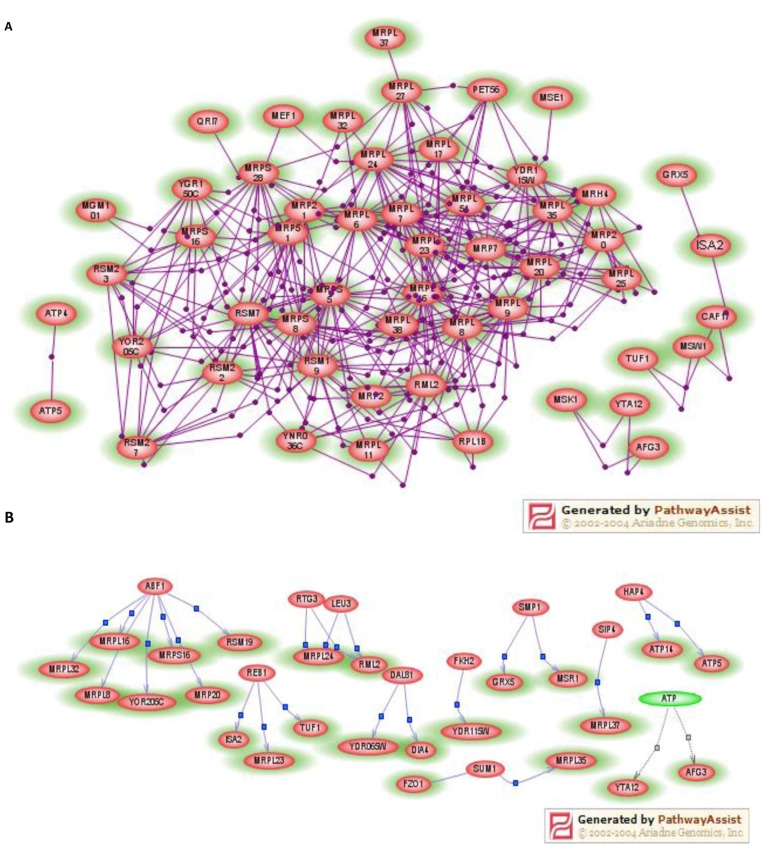
Interactions between the genes identified in this study and others using Pathway Assist. The genes loaded from the current study are shown as light green ovals with pink centers, and those shown as pink or green ovals are genes or small molecule considered as common regulators derived from the database. (A) Direct binding relationship among 53 loaded genes is shown, and lines depict direct binding relationships. Of the 53 such genes, 34 were the components of the mitochondrial large or small ribosomal protein constituents. (B) Common regulation relationship among 24 loaded genes and 11 molecules, common regulators found in the database, is displayed. Arrows depict the genes regulated by the regulators.

In an attempt to identify the genes whose inactivation leads to cell death when mtDNA was depleted we screened for 466 mutants strains for viability on YPD. Among the strains we screened we identified two novel genes encoding Atp11p and Atp12p, which are clearly required for growth of the strains devoid of mtDNA. Atp11p and Atp12p are both molecular chaperones required for the assembly of β and α subunits into the F1 sector of mitochondrial F1F0 ATP synthase, respectively [34], [35]. Human homologs of these proteins were also identified recently and they act in a manner analogous to their yeast counterpart. Moreover, human ATP12 cDNA complements yeast *ATP12Δ* disruption mutant. Both the *ATP11* and *ATP12* genes are broadly conserved in eukaryotes and are expressed in a wide range of tissues [35], [36]]. More recently, a human respiratory chain complex V deficiency was attributed to a mutation in the assembly gene *ATP12* [37]. In addition, the viability of *vps36Δ* mutant was also found to be dependent on mtDNA as shown in (**Figure 2**). However, depletion of mtDNA in vps36A background led to only reduced growth and not the complete lack of growth as found in case of Atp11 and 12 mutants. *VPS36* gene encodes a component of the ESCRT-II complex, which is involved in ubiquitin-dependent sorting of proteins into the endosome. We identified four other genes whose inactivation leads to synthetic lethality in strains devoid of mtDNA. These include *CDC73, NTA1, BZZ1* and TIM18. CDC73 is described to be a component of the Paf1p complex, binds to and modulates the activity of RNA polymerases I and II and is involved in modification of some histones, and telomere maintenance. *NTA1 is an amidase which* removes the amide group from asparagine and glutamine residues to generate proteins with N-terminal aspartate and glutamate residues. These proteins are described to be targets of ubiquitin-mediated degradation. BZZ1p regulates actin polymerization. TIM18p is a subunit of the mitochondrial TIM22 complex which mediates the assembly and stability of the complex.

### Human orthologs of yeast genes and their involvement in mitochondrial diseases

When we extended our screen to humans, we found 49 human orthologs of the identified 102 yeast genes whose absence led to a complete loss of mtDNA. Of these, 7 were genes known to be involved in mitochondrial related diseases. They are the genes encoding MutS, E. coli homolog associated with deficiency of Endometrial carcinoma; DNA Polγ involved in deficiency of progressive external ophthalmoplegia and male infertility; protoporphyrinogen oxidase associated with deficiency of porphyria variegate; ATPase family gene 3-like 2 associated with deficiency of hereditary spastic paraplegia; and three mitochondrial ribosomal proteins, L3, S12 and S14, which are linked to moebius syndrome 2, and deafness, autosomal dominant nosyndromic sensorineural 4 and 7, respectively (Table 6) [38]–[41]

**Table 6:** Genes involved in human mitochondrial disorders.

Yeast gene	Human gene				Disorder
	Symbol	Location	MIM	Title	
*MRPL9*	*MRPL3*	3q21-q23	607118	Mitochondrial ribosomal protein L3	Moebius syndrome 2
*YNR036C*	*MRPS12*	19q13.1-q13.2	603021	Mitochondrial ribosomal protein S12	DFNA4^a^
*MRP2*	*MRPS14*	1q23-1q25	601412	Mitochondrial ribosomal protein S14	DFNA7^b^
*MRPS16*	*MRPS16*	10q22.1	609204	Mitochondrial ribosomal protein S16	NLAACCFM^c^
*MSH1*	*MSH3*	5q11-q12	600887	MutS, E. coli, homolog of	Endometrial carcinoma
*MIP1*	*POLG*	15q25	174763	Polymerase, DNA, gamma	PEO^d^ and MI^e^
*HEM14*	*PPOX*	1q22	600923	Protoporphyrinogen oxidase	Porphyria variegata
*YTA12*	*AFG3L2*	18p11	604581	ATPase family gene 3-like 2	HSP^f^

a,b Deafness, autosomal dominant nosyndromic sensorineural 4 and 7, respectively.

c Neonatal lactic acidosis, agenesis of the corpus collosum and facial malformation.

d Progressive external ophthalmoplegia.

e Male infertility.

f Hereditary spastic paraplegia.

### Genetic interaction among the identified nuclear mtDNA depletor genes

**Figure 3:**
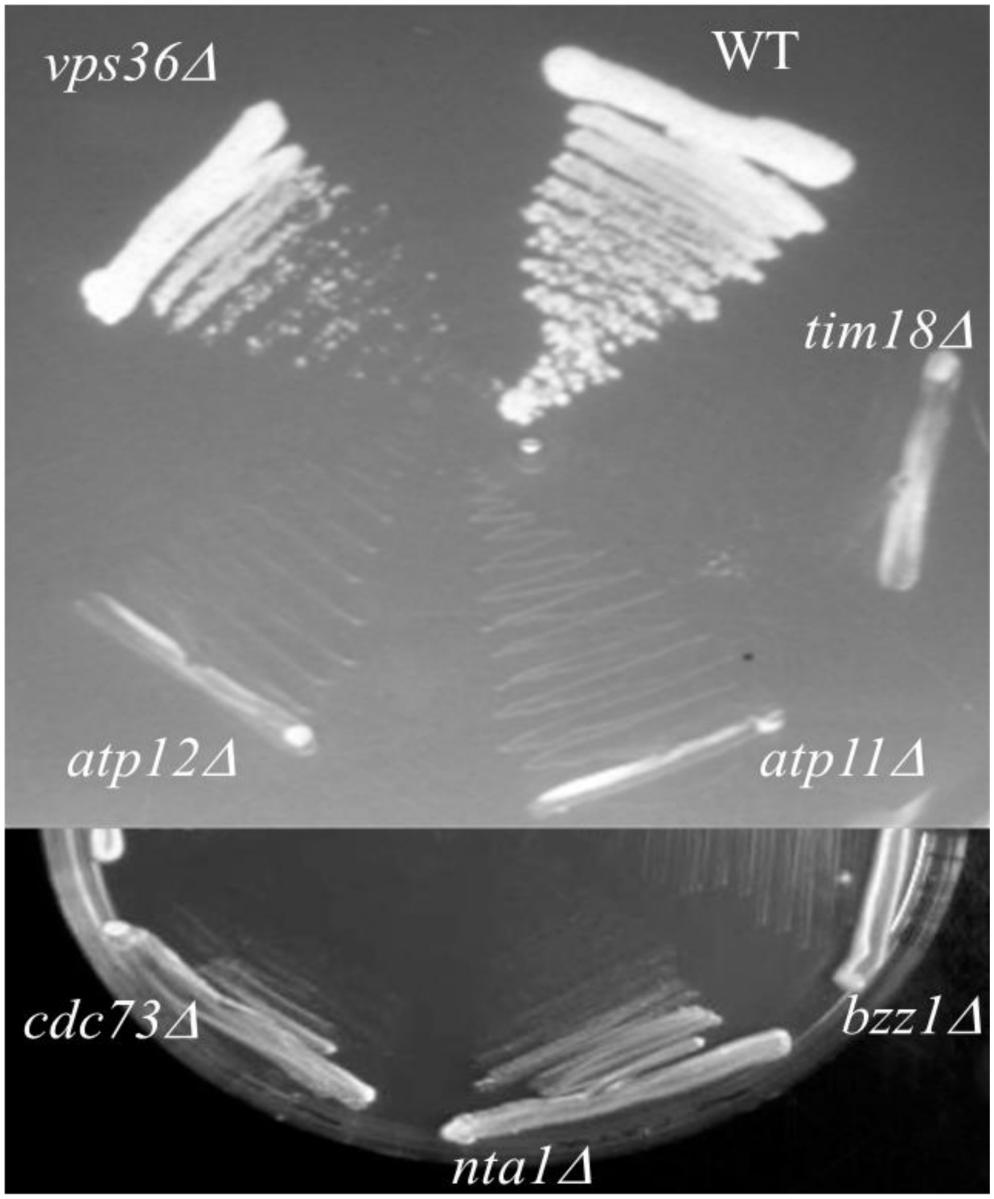
Mitochondrial DNA dependence of yeast strain viability. *atp11Δ, atp12Δ, vps36, BY4741(wt)* and *tim18Δ* (positive control) (DUNN and JENSEN 2003) strains were streaked out onto YPD plates containing 25 µg/ml ethidium bromide and allowed to grow for 3 days at 30°C to induce loss of mtDNA. To identify strains killed by the EtBr treatment, cells from YPD-EtBr plates were streaked to YPD plates lacking EtBr, and allowed to grow at 30°C for 3 days.

All 102 identified genes were loaded into Pathway Assist. Of those genes, 77 were recognized by the program (Figure 3), and were thus subjected to subsequent analysis. The ‘Find Only Direct Interaction’, ‘Find Common Regulators’ and ‘Find Common Targets’ features of the software were used to build a network of connections starting with the 77 genes. As a result, 53 genes were shown to have direct and multiple binding relationships, including 34 genes to encode mitochondrial large or small subunit ribosomal proteins and 19 genes encoding protein products involved in mitochondrial aminoacyl-tRNA synthetase, intermembrane space protein import, assembly of iron-sulfur centers or nucleic acid metabolism. The remaining 24 genes recognized by the program were found to be regulated by 11 common regulators including the genes *ABF1, REB1, RTG3, LEU3, DAL81, FKH2, SMP1, SUM1, SIP4, HAP4* and a small molecule ATP. Each common regulator was found in the database and regulates 2 or more loaded genes. These common regulators function as transcription factors to regulate expression of the handful of yeast genes. It should be pointed out that we found 2 human orthologs, *FCMD* and *KLF8*, of the common regulators *LEU3* and *SUM1* respectively, associated with human disorders. The protein encoded by *FCMD* gene regulates the migration and assembly of neurons during cortical histogenesis, and mutations in this gene may lead to Fukuyama congenital muscular dystrophy [42]. Abnormal expression of the *KLF8* has been implicated in mental retardation [43].

### Increased frequency of nuclear genome mutation in strains defective in mitochondrial function

Our previous studies suggest that disruption of OXPHOS function due to depletion of mtDNA leads to mutation in nuclear genome. We therefore determined the frequency of mutation in all 466 mutants. Our studies reveal that higher frequency of nuclear mutation with respect to the wild-type was observed in 18 out of 466 mutant strains screened, suggesting that these genes play a role in the maintenance of normal nuclear genome integrity (Table 7). Together, these studies identify key mitochondrial metabolic pathways which play important roles in protecting the integrity of nuclear genome.

**Table 7:** Frequency of cananvinine gene mutation in yeast deletion strains with mitochondrial dysfunction.

Gene deleted	Can^r^ frequency (×10^−8^) (mean ± SEM)	Wild-type function
BY4741 (WT)	122.0±10.4*	
*MHR1*	2644.9±245.7*	Protein involved in mitochondrial homologous DNA recombination and in transcription regulation
*YNL136W*	3901.9±957.9*	Subunit of the NuA4 histone acetyltransferase complex molecular function unknown
*MLH1*	8095.8±650.0*	Required for mismatch repair in mitosis and meiosis
*MSU1*	4099.9±556.5*	Mitochondrial 3′-5′ exonuclease for RNA 3′ ss-tail
*MRPS28*	565.5±98.2*	Mitochondrial ribosomal protein of the small subunit, protein biosynthesis
*YOR022C*	307.4±61.4*	Mitochondrial, biological process unknown
*FZO1*	364.5±54.1*	Mitochondrial integral membrane protein involved in mitochondrial fusion and maintenance of the mitochondrial genome
*COX20*	472.1±89.7*	Mitochondrial inner membrane protein, required for proteolytic processing of Cox2p and its assembly into cytochrome c oxidase
*YDR512C*	640.1±57.3*	Molecular function unknown
*VMA21*	2396.6±394.4*	Vacuolar acidification
*PET309*	428.1±67.4*	Required for stability and translation of *COX1* mRNA
*MEF1*	315.4±22.1*	Mitochondrial elongation factor G-like protein
*YLR091W*	398.5±54.6*	Molecular function unknown
*RSM22*	714.4±108.1*	Mitochondrial ribosomal protein of the small subunit
*PIF1*	1345.8±55.7*	DNA helicase involved in mitochondrial DNA repair and telomere/DNA recombination
*ABF2*	1478.6±161.0*	Mitochondrial DNA-binding protein involved in mitochondrial DNA replication and recombination
*YER087W*	435.7±88.6*	Proline-tRNA ligase activity

* p<0.05.

## Discussion

To gain insights into the genetic contribution of nuclear genes to maintaining mitochondrial genome stability, we have taken a systematic approach to the identification of nuclear genes by screening a set of yeast deletion strains demonstrating a complete loss of mtDNA. Our screen led to the identification of 102 such genes, which may be classified into the following five categories. The first category includes 56 genes involved in the mitochondrial protein synthesis. The second category consists of 15 genes related to nucleic acid metabolism. The third category is composed of 4 genes required for mitochondrial OXPHOS. The fourth category is 14 genes required for various other biological processes such as bud-site selection, mitochondrial intermembrane space protein import, assembly of cytochrome-c oxidase, vacuolar protein sorting, protein-nucleus import, calcium-mediated signaling, heme biosynthesis or iron homeostasis. The final category includes 13 genes that have not been well defined for the mechanism by which they affect mtDNA maintenance. Of the 102 genes identified, 52 were not reported previously. Our work therefore provides a new set of nuclear genes tightly controlling mtDNA copy number in yeast cells.

It is striking that over 50% of the identified mutants completely lost their mtDNA due to a defect in mitochondrial protein synthesis. The phenotype suggests that the wild-type genes of these nuclear mutants play crucial roles in the mtDNA maintenance by governing mitochondrial protein translation system. Consistent with this observation, an earlier study showed that yeast mutant strains, totally blocked in mitochondrial protein synthesis due to a disruption of genes coding for either mitochondrial aminoacy-tRNA synthestases, an elongation factor, or a putative protein of mitochondrial ribosomes, undergo a rapid quantitative conversion to rho^−^ derivatives [44]. Another study also found that growth of yeast in the presence of inhibitors of mitochondrial protein synthesis induces a high frequency of rho^−^ [45]. Our study thus provides strong support for the notion that mitochondrial translation is required for the maintenance of mitochondrial genome stability. A dysfunction in the synthesis of mitochondrial ribosomal protein components appears to be a primary cause leading to the observed loss of mtDNA. In our observations, 38 out of the 56 protein synthesis-deficient strains with a disappearance of mtDNA (67.9%) are dysfunctional in synthesizing the mitochondrial large or small ribosomal subunit proteins. This is conceivable considering that the ribosome, consisting of rRNA species and MRPs, plays a central role in protein translation process. In yeast, mitochondrial ribosome contains at least 90 proteins encoded by the nuclear genome [46]. They are normally synthesized in cytoplasm and transported into the mitochondria, where they are assembled into large or small ribosomal subunit, coordinately providing a place for mitochondrial protein synthesis [47]. The close interactions between ribosomal protein constituents are also revealed by the PathwayAssist analysis data. The fact that direct binding relationship exists among the 53 ribosomal protein encoding genes indicates that protein products of these genes interact actively. Alternatively, the observed loss of mtDNA in protein synthesis-deficient strains may be a secondary effect caused by the absence of a specific aminoacyl-tRNA synthetase responsible for activating an amino acid to be incorporated into a protein chain, or some other proteins products involved in mitochondrial ribosome recycling or assembly, mitochondrial translation elongation, peptide chain release and translation-required GTPase or GTP binding protein. Altogether, these results indicate that defects of nuclear genes involved in the mtDNA translational process are a major cause leading to the disappearance of mtDNA in yeast, opening new avenues of investigation toward understanding the role of mitochondrial dysfunction in human disease and calling for more attention and studies in this area.

A set of complete mtDNA loss in a handful of yeast mutants was caused by other defects than mitochondrial protein translation process as shown in Table 2–4. This include 15 mutants (14.7%) defective in nucleic acid metabolism such as RNA polymerase synthesis, DNA binding, DNA replication, one-carbon compound metabolism and RNA or DNA helicase. Expectedly, one of these strains is the *MIP1* mutant. This gene encodes a 140-kDa polypeptide with both polymerization and 3′→5′ exonuclease proofreading activities, playing an essential role for the replication of mtDNA. In agreement with our observation, a few prior studies showed that disruption of *MIP1* gene might reduce the mtDNA copy number in cells [48], [49]. Besides, 4 (3.9%) mutants were defective in mitochondrial oxidative phosphorylation such as ATP synthesis and iron-sulfur center assembly; 14 (13.7%) mutants defective in the processes like bud-site selection, mitochondrial protein import, assembly of cytochrome-c oxidase, protein sorting, sphingolipid metabolism, protein folding and small molecule metabolism. Additionally, 13 mutants (12.7%) have deletions of genes whose functions are still unknown so far. These striking multiple causes resulting in a complete loss of mtDNA suggest that apart from the mitochondrial protein synthesis process, many other nuclear-encoded factors are also involved in the mtDNA maintenance.

When we extended our screen to humans, we found 47 human orthologs of the 102 identified yeast genes whose deletions led to the complete loss of mtDNA. Of these orthologs, 8 were already found to be involved in human mitochondrial diseases. These include genes encoding four mitochondrial ribosomal proteins, Pol γ, protoporphyrinogen oxidase, ATPase and a protein associated with DNA mismatch repair (Table 6). The corresponding diseases are Moebius syndrome 2; deafness, autosomal dominant nosyndromic sensorineural 4 and 7, respectively; neonatal lactic acidosis, agenesis of the corpus collosum and facial malformation; progressive external ophthalmoplegia and male infertility; porphyria variegata and hereditary spastic paraplegia endometrial carcinoma. In light of the high similarity between yeast and human mitochondria, although other 39 orthologs were not suggested to have association with human disease yet, but it is possible that a link might be found in the future between these human genes and a disease since many human disease genes are associated with wide spectrum of yeast deletion phenotypes [50]. Especially, 8 of the 39 orthologs encode human mitochondrial ribosomal proteins. The implication of this finding is that these proteins are responsible for translating the 13 mRNAs for essential proteins of the OXPHOS, and therefore are candidates for involvement in human genetic diseases. Recently, a new group of mitochondrial ribosomal genes has been proposed as candidates genes for human mitochondrial disorders such as Leigh Syndrome, multiple mitochondrial dysfunctions and non-syndromic hearing loss [51]. In addition, this study showed that 2 of the 11 common regulators displayed by Pathway Assist program, *LEU3* and *SUM1*, have their human orthologs, *FCMD* and *KLF8*, which are associated with human disorders [52], [53], providing new facet of nuclear gene involvement in mitochondrial related human disorders.

To study the effect of different gene deletions on nuclear genome integrity, we measured the frequency of nuclear point mutations using the *CAN1*-forward mutation. Canavanine is lethal to yeast cells and enters the cell *via* the arginine permease, encoded by the *CAN1* gene. Cells that can synthesize arginine and acquire a mutation in *CAN1* gene are therefore able to grown on medium lacking arginine and containing canavanine. We found that the deletion of each of seventeen nuclear genes resulted in higher frequency of the mutations with respect to the wild-type, suggesting that these genes are important to maintain nuclear genome stability.

In conclusion, we have identified a set of nuclear genes whose absence lead to a loss of mtDNA, and provide new convincible evidence in elucidating how mtDNA content is maintained by nuclear genes. An increased understanding of orthologous human genes will help to determine the pathological consequences resulting from changes in mtDNA content.
